# Glucosylceramide synthase inhibition reduces ganglioside GM3 accumulation, alleviates amyloid neuropathology, and stabilizes remote contextual memory in a mouse model of Alzheimer’s disease

**DOI:** 10.1186/s13195-022-00966-0

**Published:** 2022-02-01

**Authors:** James C. Dodge, Thomas J. Tamsett, Christopher M. Treleaven, Tatyana V. Taksir, Peter Piepenhagen, S. Pablo Sardi, Seng H. Cheng, Lamya S. Shihabuddin

**Affiliations:** 1 Rare and Neurological Diseases Therapeutic Area, Sanofi, 49 New York Avenue, Framingham, MA 01701 USA; 2 Translational In Vivo Models, Sanofi, 5 Mountain Road, Framingham, MA 01701 USA

**Keywords:** Glycosphingolipids, Glucosylceramide, Dementia, Cortex, Hippocampus, Amygdala

## Abstract

**Background:**

Gangliosides are highly enriched in the brain and are critical for its normal development and function. However, in some rare neurometabolic diseases, a deficiency in lysosomal ganglioside hydrolysis is pathogenic and leads to early-onset neurodegeneration, neuroinflammation, demyelination, and dementia. Increasing evidence also suggests that more subtle ganglioside accumulation contributes to the pathogenesis of more common neurological disorders including Alzheimer’s disease (AD). Notably, ganglioside GM3 levels are elevated in the brains of AD patients and in several mouse models of AD, and plasma GM3 levels positively correlate with disease severity in AD patients.

**Methods:**

Tg2576 AD model mice were fed chow formulated with a small molecule inhibitor of glucosylceramide synthase (GCSi) to determine whether reducing glycosphingolipid synthesis affected aberrant GM3 accumulation, amyloid burden, and disease manifestations in cognitive impairment. GM3 was measured with LC-MS, amyloid burden with ELISA and amyloid red staining, and memory was assessed using the contextual fear chamber test.

**Results:**

GCSi mitigated soluble Aβ42 accumulation in the brains of AD model mice when treatment was started prophylactically. Remarkably, GCSi treatment also reduced soluble Aβ42 levels and amyloid plaque burden in aged (i.e., 70 weeks old) AD mice with preexisting neuropathology. Our analysis of contextual memory in Tg2576 mice showed that impairments in remote (cortical-dependent) memory consolidation preceded deficits in short-term (hippocampal-dependent) contextual memory, which was consistent with soluble Aβ42 accumulation occurring more rapidly in the cortex of AD mice compared to the hippocampus. Notably, GCSi treatment significantly stabilized remote memory consolidation in AD mice—especially in mice with enhanced cognitive training. This finding was consistent with GCSi treatment lowering aberrant GM3 accumulation in the cortex of AD mice.

**Conclusions:**

Collectively, our results indicate that glycosphingolipids regulated by GCS are important modulators of Aβ neuropathology and that glycosphingolipid homeostasis plays a critical role in the consolidation of remote memories.

**Supplementary Information:**

The online version contains supplementary material available at 10.1186/s13195-022-00966-0.

## Background

Gangliosides are a subgroup of glycosphingolipids that have important roles in regulating several aspects of cellular function including membrane curvature, receptor localization and signaling, calcium homeostasis, autophagy, and apoptosis [47]. The synthesis of the gangliosides (e.g., GM3, GM2, and GM1) occurs with the successive addition of galactose and sialic acid moieties to glucosylceramide (GlcCer). Gangliosides are highly abundant in the CNS and are essential for its normal development and maturation; however, in some rare inherited lysosomal storage disorders (e.g., Sandhoff disease), aberrant ganglioside accumulation is pathogenic. Neuropathological and clinical phenotypes associated with ganglioside storage include neurodegeneration, neuroinflammation, demyelination, seizures, hearing loss, motor impairment, and dementia [3, 41, 43].

Several lines of evidence suggest that more subtle ganglioside accumulation also contributes to the pathogenesis of Alzheimer disease (AD), the most common form of dementia. For example, GM3 and GM1 levels are elevated in the brains of AD patients [9, 24, 36, 42] and in AD rodent models [7, 8, 27, 54]. GM1 bound amyloid beta (Aβ) is found in the brains of AD and Down syndrome patients, but not in normal individuals [52, 53]. Moreover, two large clinical studies examining the peripheral lipidome of AD patients concordantly showed that GM3 levels were strongly associated with disease [22]. Gangliosides are reported to promote the amyloidogenic processing of amyloid precursor protein (APP), Aβ aggregation, and subsequently the development of amyloid plaques [10, 23, 32, 34, 51–53]. Ganglioside bound Aβ undergoes a conformational transition from a random coil to a toxic β-sheet structure that acts as a seed (i.e., a polymerizer) to accelerate amyloid fibril formation [10, 23, 32]. In addition, reducing ganglioside levels alleviates Aβ toxicity in preclinical AD models. Notably, inhibiting the synthesis of glucosylceramide (GlcCer), a ganglioside precursor, reduces Aβ generation and neurotoxicity in vitro [18, 38, 49]. Furthermore, cross-breeding mice deficient in either GlcCer synthase or GM3 synthase with AD model mice mitigates the manifestation of Aβ-associated neuropathology and cognitive deficits [4, 17, 21]. It remains unknown, however, whether reducing ganglioside levels in symptomatic AD mice with pre-existing Aβ accumulation will slow disease pathogenesis.

In contrast to the reported benefits found with ganglioside lowering in AD model systems, a limited number of studies also suggest that ganglioside augmentation may be neuroprotective in AD. For example, ganglioside treatment mitigated toxicity triggered by fibrillar Aβ in organotypic hippocampal slice cultures [29], reduced Aβ plaque deposition, tau phosphorylation, and cognitive dysfunction in AD model mice [14, 45], and appeared to slow further deterioration in a small number of AD patients with early-onset disease [48].

Here, we determined whether lowering glycosphingolipid levels in transgenic mice carrying the human APP695 Swedish mutation (Tg2576 mouse model) with a CNS penetrant inhibitor of GlcCer synthase (GCSi) affected disease progression. We found that GCSi treatment in Tg2575 mice reduced brain GM3 levels to WT levels, lowered soluble Aβ42, which is particularly toxic to neurons [13] and a determinant of disease severity in AD patients [33], and improved remote memory consolidation. Notably, reductions in Aβ42 neuropathology and amyloid burden occurred even when GCSi treatment was initiated in aged Tg2576 mice. Collectively, our findings suggest that glycosphingolipids are significant modulators of AD relevant neuropathology and cognition.

## Methods

### Experimental design and statistics

The experimental design for each assay is described in its subsection below. Normality was determined using the Shapiro-Wilk normality test. Data sets that failed the normality test were analyzed with a Mann-Whitney test. A two-tailed unpaired *t* test was used to compare data sets that passed the normality test and that had equal variances. If data set variances were significantly different, then an unpaired *t* test with Welch’s correction was used to compare groups that passed the normality test. Statistical tests comparing multiple groups were performed using a one-way analysis of variance (ANOVA) followed by a Dunnett’s multiple comparison post hoc test to find differences between group means. A value of *p*<0.05 was considered statistically significant. All statistical tests were performed using GraphPad Prism Software 8.0.

### Animals and glucosylceramide synthase inhibitor (GCSi) treatment

Cohorts of Tg2576 mice that express mutant APP (APP695SWE) and WT controls were purchased from Taconic Bioscience (Rensselaer, NY) for each experiment (supplementary Table I). Animals were housed under light: dark (12:12 h) cycles and provided with food and water ad libitum. In all GCSi treatment studies, compound (GENZ-667161) was administered at 60 mg/kg/day in formulated diet as previously described [5, 16, 31, 44]. All procedures were performed using protocols approved by Sanofi Genzyme’s Institutional Animal Care and Use Committees.

### Fear conditioning

Mice were trained in four of the Med Associates (Fairfax, VT) Near Infrared Fear Conditioning System chambers. In characterization studies, mice were trained either at 12, 20, or 36 weeks of age (*N*=12/sex/genotype/training age). Mice used in the testing of GCSi were trained at 12 weeks of age (*N*=10/sex/treatment). During the training session, mice were placed in the contextual fear chamber in “Context A,” which consisted of lighting, a neutral background, and a stainless-steel grid floor, and were then exposed to a two-trial delay-cued protocol. Briefly, mice were given 2 min to explore the chamber in Context A before a conditioned stimulus (CS) of a 2000-Hz cue was given. Thirty seconds later, a 1-s unconditioned stimulus (US) of 1.2-mA foot shock was applied. With an intertrial interval (ITI) of 60 s, the US-CS pairing was then repeated. After a 24-h retention period, mice were brought back to the testing room. After a 1-h habituation period, mice were placed back in Context A for 5 min to measure freezing behavior. Freezing (defined as the lack of movement, except for respiration) was recorded using a near-infrared camera system. Mice were then removed from the chamber and placed back into their respective cages. After 1 h, mice were placed back into the chamber in a novel context, Context B to test cued memory. During the test mice were allowed to explore the cage for 2 min in the novel environment, followed by 3 min of the two-tone auditory cue with the same ITI as the training protocol. Again, freezing to the novel environment and the cue were assessed with the near infrared camera system. Approximately 8 and 16 weeks after the initial training session, remote contextual memory consolidation was tested. Mice were habituated to the testing room for 1 h and were then placed in Context A for 5 min to measure freezing behavior. Retrained (RT) mice were initially exposed to the protocol described at 12 weeks of age and then again at 36 weeks of age. Mice that underwent the more intense training (MIT) protocol were exposed to four US-CS pairings at 36 weeks of age (*N*=12/sex/genotype), each with an ITI of 60s. Contextual memory is defined as the freezing from the training context minus the freezing in the novel context. Cued memory is defined as the freezing to the CS in the novel context.

### Glycosphingolipid analysis

Glycosphingolipids were measured in brain tissue samples by liquid chromatography coupled with tandem mass spectrometry (LC/MS/MS) as described in our previous studies [5, 16, 31, 37, 44]. Tg2576 mice were fed either a standard diet or diet formulated with GCSi from 12 to 68 weeks. At sacrifice brains from PBS perfused Tg2576 and age-matched WT mice (*N* = 4/sex/group) were rapidly removed, flash-frozen, and stored at −80C until lipid extraction. Tissue pieces (~ 50 mg) were homogenized using a Qiagen bead beater for 10 min at 30 Hz in an 80/15/5 (v/v/v) mix of methanol, acetonitrile, and HPLC grade water yielding a 25-mg/ml tissue homogenate. The samples were sonicated for 10 min and then clarified by centrifugation for 5 min at 1500 g. The samples were then diluted 1:10 with the homogenization solvent yielding a final concentration of 2.5 mg tissue/ml. The resulting solution was sonicated for 10 min and centrifuged at 3000g to pellet debris. Previously, we reported that this extraction process (in contrast to chloroform/methanol-based methods) minimizes lipid loss (< 5% of total glycosphingolipids) and improves lipid recovery reproducibility with a relative standard deviation typically below 15%. Furthermore, we also found the amount of extracted glycosphingolipid correlated linearly (*R*^2^ > 0.98) with the amounts of brain tissue sample [37]. GM3, GM2, and GM1 were measured by transferring an aliquot of the supernatant to a vial containing dried internal standards (Matreya, Inc., Pleasant Gap, PA, USA) and injecting into an Agilent 1100 HPLC system (Agilent, Palo Alto, CA) interfaced with a Qtrap 4000 mass spectrometer (AB Sciex, Toronto, Canada) system (LC/MS/MS). Chromatographic separation was achieved with a normal-phase silica column run in isocratic mode with a mixture of methanol/acetonitrile/water as the mobile phase, and MS/MS was performed in MRM mode [11]. For separation and analysis of glucosyl- and galactosylceramide, a second aliquot of the supernatant was added to a vial containing internal deuterated standards (Matreya, Inc., Pleasant Gap, PA, USA) and dried before being reconstituted in a 95/5 (v/v) mix of acetonitrile and methanol. Reconstituted extract was then injected into an LC–MS/MS system consisting of a Waters Acquity UPLC and an AB Sciex 4000Qtrap mass spectrometer. Separation of glucosylceramide and galactosylceramide was achieved through the series coupling of 2 Waters Acquity UPLC Beh Hilic Columns (2.1 × 150 mm, 1.7 μm each) under the following conditions: Mobile Phase A consisting of acetonitrile with 5 mmol/l of ammonium acetate and 0.5% acetic acid, while Mobile Phase B was composed of methanol containing those same additives. The system was run in isocratic mode for 25 min, at a composition of 99%A/1%B. The columns were held at a constant temperature of 40 °C, while the autosampler was set to 18 °C [6]. Reported glycosphingolipid levels are normalized to milligrams of the brain tissue.

### Amyloid beta (Aβ) analysis

Tg2576 mice and WT controls were sacrificed at 20 and 36 weeks of age (*N*=6/sex/genotype/age) in experiments characterizing age and sex related changes in Aβ accumulation in the brain. In GCSi experiments, Tg2576 mice were fed either a standard or GCSi formulated diet until sacrifice. Drug diet studies were carried out from 12 to 68 weeks (*N* = 6–8/group) and from 70 to 90 weeks of age (*N* = 6–8/group). At sacrifice brains from PBS perfused mice were rapidly removed, flash-frozen in liquid nitrogen, and then stored at −80C until they were blunt dissected to prepare sample homogenates containing the cortex, hippocampus, and amygdala. Brain samples were sequentially extracted in a two-step extraction: sonication in (1) 2% SDS and (2) 70% formic acid (FA) as previously described [26] with the latter designated as the insoluble fraction. After sonication, the samples were centrifuged at 100,000×*g* for 1 h at 4°C, the supernatant was recovered, and the pellet was sonicated with the next solution. Brain extracts were measured for Aβ40 and Aβ 42 by sandwich ELISA (Covance, SIG-38954, SIG-38956) according to the manufacturer’s instructions. Softmax Pro software (Molecular Devices, San Jose, CA) was used to calculate femtomoles per milliliter by comparing the sample absorbance to the absorbance of known concentrations of synthetic Aβ1–40 and Aβ1–42 in identical solution as the samples, and these values were corrected with the wet weight of the original homogenate to be finally expressed as picomoles per gram wet weight.

### Amyloid red staining and plaque analysis

Tg2576 mice were fed either a standard diet or diet formulated with GCSi (*N* = 4/group) from 70 to 90 weeks. At sacrifice, Tg2576 mice and age-matched WT mice (*N* = 4/group) were deeply anesthetized with euthasol (150 mg/kg delivered intraperitoneally) and then transcardially perfused with 0.1 M of phosphate-buffered saline (PBS) followed by 4% buffered paraformaldehyde. The brains were promptly removed, post-fixed in the same fixative for 48 h at 4°C and were then immersed in 30% sucrose until equilibration was reached. The brains were then cryostat-sectioned in the coronal plane at 20 μm. Amyloid stain solution was prepared by mixing 0.5 g amyloid red (Anateck LTD, MI, cat# 863), 0.3 g sodium chloride (Sigma-Aldrich, MO, cat# S7653), 49 ml hot DI water, 20 ml 0.1M dibasic sodium phosphate (J.T. Baker, NJ, cat#4062-01), 20 ml 100% ethanol, and 11 ml 0.1 N HCl (Sigma-Aldrich, MO, cat#H9892). Amyloid stain solution was warmed up in an oven until its temperature reached 50–55°C. Frozen slide sections were then hydrated in 70% ethanol for 5 min and 50% ethanol for 5 min and were then stained with amyloid red solution for 20 min at 50–55°C. Next, slides were then rinsed in DI water followed by Harris Hematoxylin (Sigma-Aldrich, MO, cat#HHS16) for 15 s and were then rinsed in tap water until blue. Finally, slides were dehydrated in 100% ethanol for 5 min, xylene (3 X 1 min) and then mounted with acrytol (Shur Mount Xylene Based, Electron Microscopy Sciences, PA, cat#17991-01). Slides (6 sections/slide) were imaged at 20X using a Mirax slide scanner (Carl Zeiss, Jena, Germany). Regions of interest (ROIs) were manually drawn around the hippocampus, dorsal subiculum, cortex, piriform cortex, and the amygdala. Images were analyzed using an automated algorithm generated with Axiovsion software (Carl Zeiss, Jena, Germany) which applied the same color threshold to identify amyloid-positive plaques in each image to quantify amyloid staining in each ROI. Data was generated for total amyloid area, amyloid fraction (amyloid area/region area), and the number of plaques in each region.

## Results

### Impaired remote memory stabilization precedes short-term memory deficits in Tg2576 mice

**Fig. 1:**
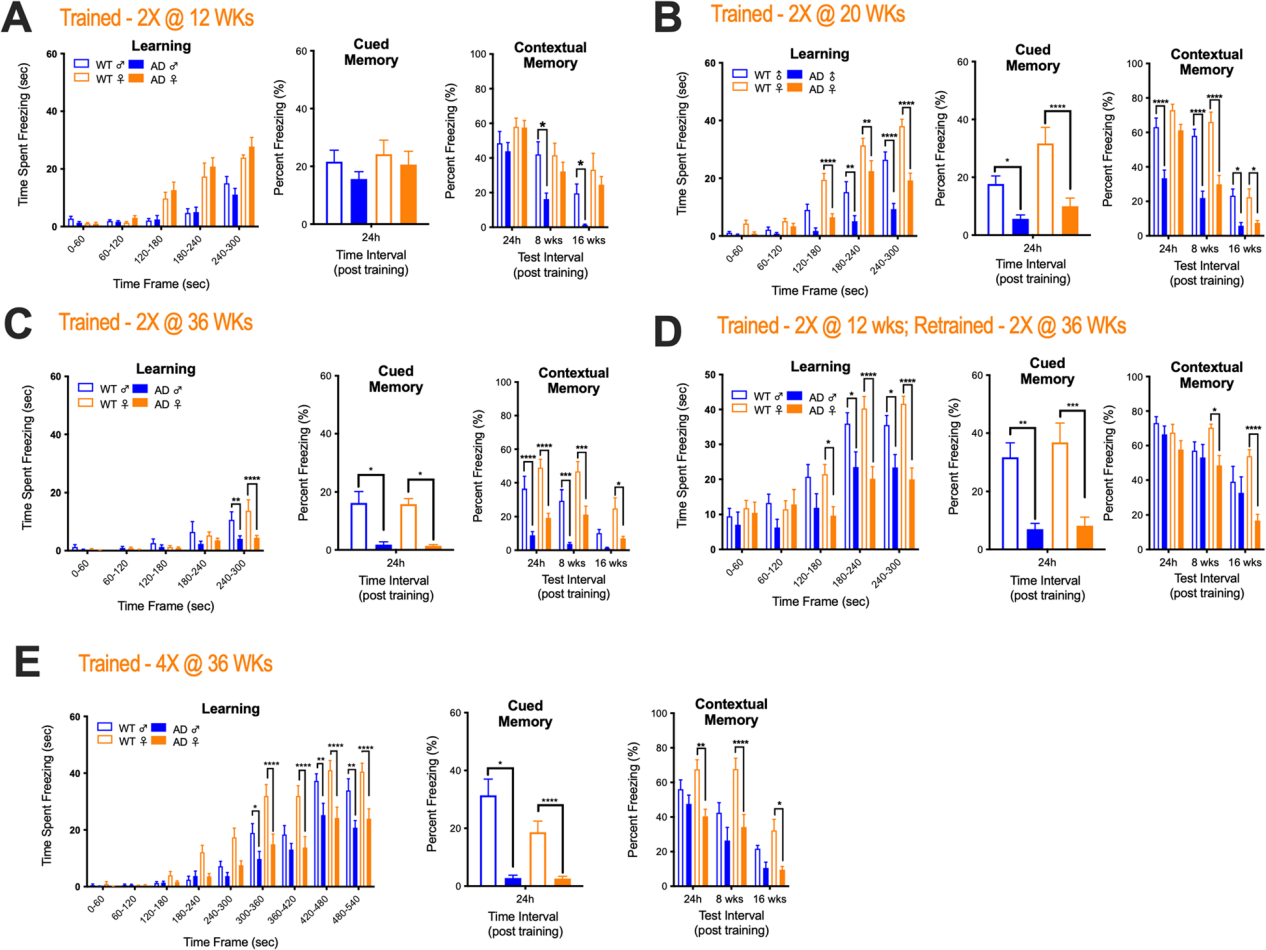
Alzheimer disease (AD) model mice display age- and sex-related differences in cognitive phenotypes. Separate cohorts of AD (Tg2576) model mice were tested at **A** 12, **B** 20, and **C** 36 weeks of age for sex- and age-related deficits in learning, cued (amygdala-dependent) memory, contextual (hippocampal-dependent) short-term memory, and remote (cortical-dependent) memory consolidation using the contextual fear chamber conditioning test. Mice in cohorts **A**–**C** were exposed to two conditioned stimulus (CS; 2000-Hz cue) and unconditional stimulus (US; 1.0-mA foot shock) pairings during training (Trained -2X). Additional cohorts of mice (**D**, **E**) were tested for cognitive impairment after enhanced cognitive training. Mice in **D** were exposed to two CS-US pairings at 12 weeks of age and were then retrained at 36 weeks of age with 2 additional CS-US pairings, where mice in E received four CS-US pairings at 36 weeks of age during training (Trained 4X). Statistical comparisons for AD mice are compared to sex matched wild type (WT) controls (****p = 0.0001, ***p = 0.001, **p = 0.01, and *p = 0.05). Error bars represent ± SEM

Prior to testing the therapeutic efficacy of lowering ganglioside levels in the Tg2576 mouse model of AD, we characterized separate cohorts of mice at 12, 20, and 36 weeks of age for sex and age-related deficits in learning, cued (amygdala-dependent) memory, contextual (hippocampal-dependent) short-term memory, and remote (cortical-dependent) memory consolidation using the contextual fear chamber conditioning test. At 12 weeks of age learning, cued memory and short-term contextual memory were equivalent between Tg2576 and WT mice. Remote memory consolidation, however, was significantly impaired in male, but not in female Tg2576 (Fig. 1A). At 20 weeks of age, learning and cued memory were adversely affected in Tg2576 mice regardless of sex; however, only male Tg2576 mice displayed significantly impaired contextual short-term memory. In contrast, remote memory consolidation was significantly impaired in both male and female Tg2576 mice (Fig. 1B). Interestingly, despite sex-differences in cognitive behavior we did not find any sex-differences in the rate of Aβ species accumulation in the cortex, hippocampus, or amygdala of Tg2576 at either 20 or 36 weeks (Fig. S1). However, regional variation in the rate of Aβ species accumulation was observed with the cortex showing the most rapid rate of accumulation, which is consistent with cortical dependent memories being adversely affected first in this AD mouse model (Fig. S1). At 36 weeks of age, learning was adversely affected in WT (compared to 12- and 20-week WT old mice) and in Tg2576 mice. Female Tg2576 mice also now featured significant short-term contextual memory impairments that were like their male diseased counterparts. Like mice trained at 20 weeks of age, cued and remote memory consolidation were significantly impaired in Tg2576 mice trained at 36 weeks of age (Fig. 1C).

Next, we sought to evaluate cognitive function in 36-week-old mice that were either re-trained (RT) or exposed to a more intense training (MIT) procedure to determine if memory deficits still manifested in aged mice with enhanced cognitive training. Mice in the RT cohort were trained at 12 weeks of age and then again at 36 weeks of age (i.e., mice were exposed to 2 CS-US pairings per training session, see the “Methods” section for details). Mice in the MIT cohort were exposed to 4 CS-US pairings at 36 weeks of age. Learning and cued memory were still significantly impaired in RT and MIT Tg2576 mice regardless of sex when compared to similarly trained WT counterparts (Fig. 1D, E). Notably, however, male Tg2576 mice that underwent either RT or MIT now displayed short-term contextual and remote memory consolidation that was comparable to WT mice (Fig. 1D, E). Interestingly, in female RT Tg2576 mice short-term contextual memory was also now like WT mice, but remote memory consolidation was still significantly impaired (Fig. 1D). In contrast, MIT exposure did not improve either short-term contextual or remote memory consolidation in female Tg2576 mice (Fig. 1E). Collectively, our findings indicate that remote memory consolidation deficits precede learning and short-term memory impairments in Tg2576 mice. This observation is at odds with what is typically found in AD patients, where deficits in short-term memory typically manifest prior to impairments in long-term memory [2].

### Inhibition of glucosylceramide synthase reduces glycosphingolipid levels in Tg2576 mice

**Fig. 2:**
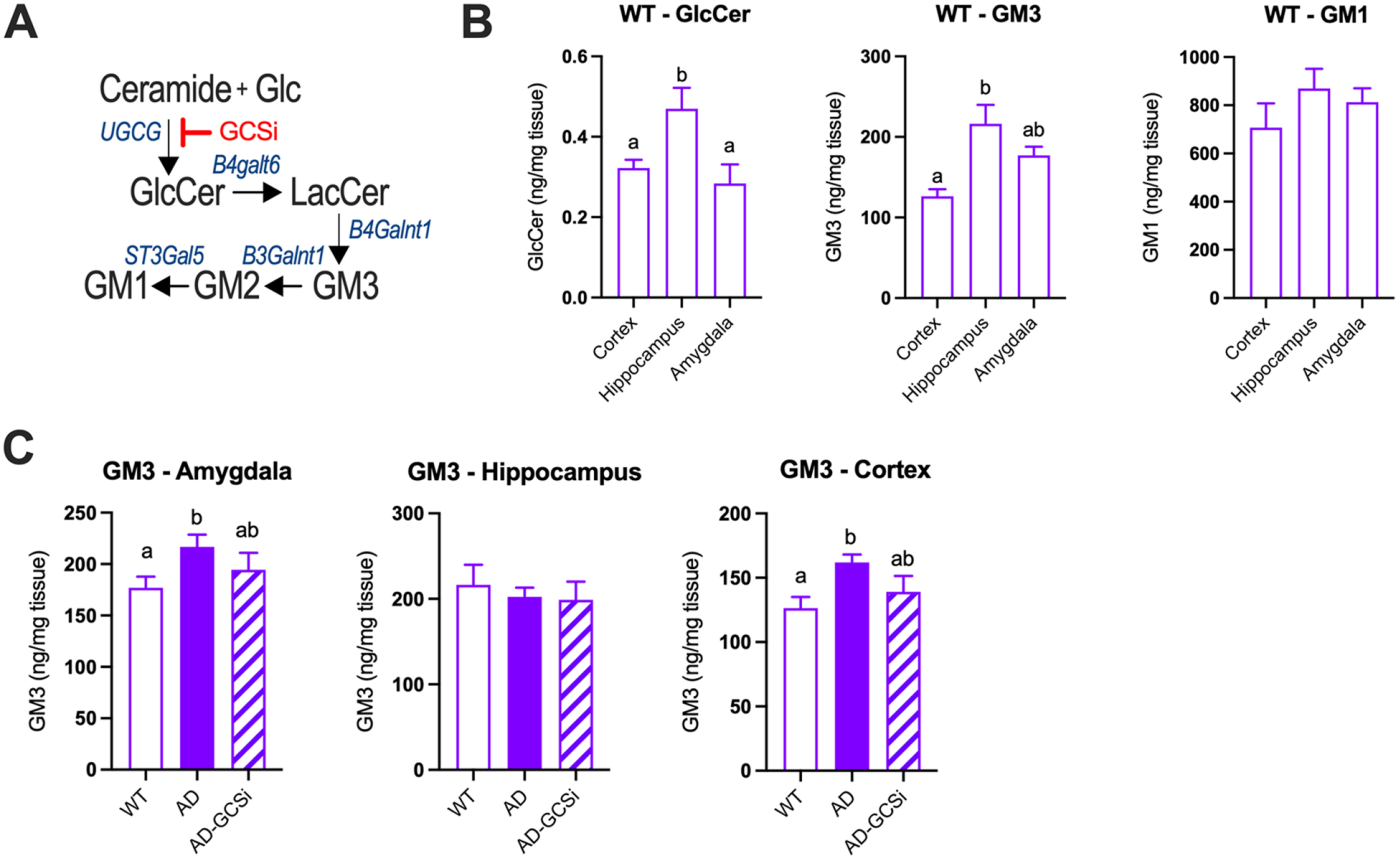
Brain ganglioside GM3 levels in AD mice are reduced after glucosylceramide (GlcCer) synthase inhibitor (GCSi) treatment. **A** Overview of the glycosphingolipid pathway (Glc, glucose; LacCer, lactosylceramide; GM3, GM2 and GM1, ganglioside GM3, GM2 and GM1; UGCG, GlcCer synthase; B4galT6, LacCer synthase; B4galnt1, GM3 synthase; B3galnt1, GM2 synthase; St3gal5, GM1 synthase). **B** WT mice feature regional variation in GlcCer and GM3 levels in the cortex, hippocampus, and amygdala. **C** AD (Tg2576) mice treated with GCSi show significant reductions in GM3 levels in the amygdala and cortex. Columns not connected by the same letter are significantly (*p* = 0.01) different from each other. Error bars represent ± SEM

Diseased-related changes in brain ganglioside levels are observed in several rodent AD preclinical models [7, 8, 27, 54]. Thus, we wanted to determine whether gangliosides also accumulate in the brains of Tg2576 mice and whether disease associated elevations in gangliosides are lowered after treatment with a CNS penetrant inhibitor of glucosylceramide synthase (GCSi) (Fig. 2A). Tg2576 mice were fed either a control diet or a GCSi formulated diet, which we previously reported lowers glycosphingolipid levels and reduces neuropathological features of disease in mouse models of Gaucher disease [5], Parkinson disease [44], and amyotrophic lateral sclerosis [16]. In WT mice, regional variations in total GlcCer and GM3 levels were observed in the cortex, hippocampus, and amygdala (Fig. 2B). Total GlcCer and GM1 levels were similar in the cortex, hippocampus, and amygdala of untreated Tg2576 and WT mice (Fig. S2). GCSi treatment significantly reduced total GlcCer levels in the cortex and hippocampus of Tg2576 compared to controls (Fig. S2). Total GM3 levels were significantly increased in the cortex and amygdala of Tg2576 mice compared to WT controls. GM3 levels in the cortex and amygdala of Tg2576 mice were significantly lowered with GCSi treatment (Fig. 2C). GM2 was below the limit of detection in all samples analyzed. Total GM1 levels in the brains of Tg2576 were like WT mice regardless of the brain region analyzed and were not significantly affected by GSCi treatment (Fig. S2). Collectively, our results show that Tg2576 mice display disease related changes in brain GM3 levels that are reduced with GCSi treatment.

### GCSi reduces soluble Aβ42 levels and amyloid plaque burden in aged Tg2576 mice

**Fig. 3:**
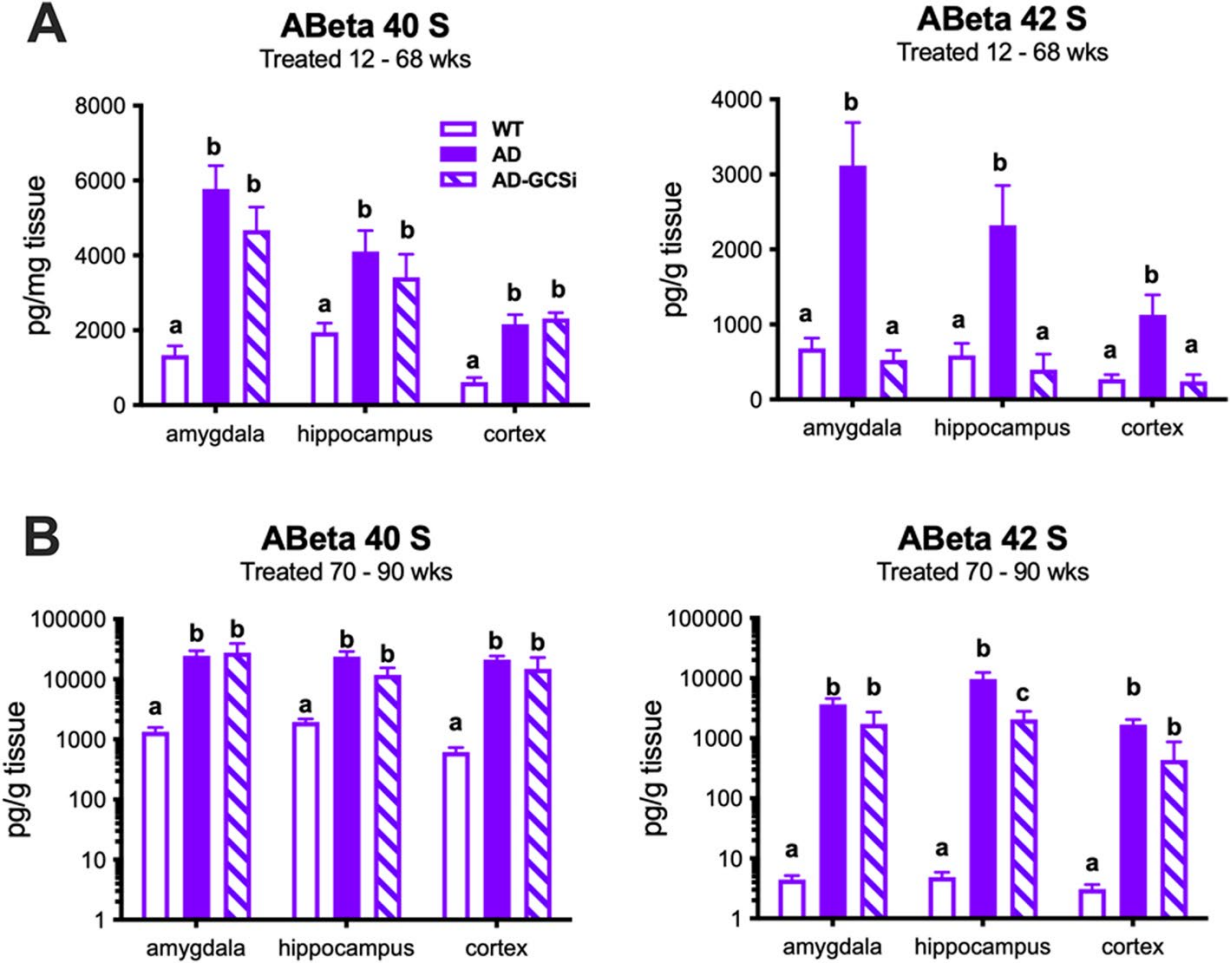
GCSi treatment reduces soluble Aβ42 accumulation in the brains of AD mice. Soluble Aβ40 and Aβ42 species in the amygdala, hippocampus, and cortex of AD (Tg2576) mice after GCSi treatment from **A** 12 to 68 weeks of age and **B** 70 to 90 weeks of age. Columns not connected by the same letter are significantly (*p* = 0.01) different from each other. Error bars represent ± SEM

**Fig. 4:**
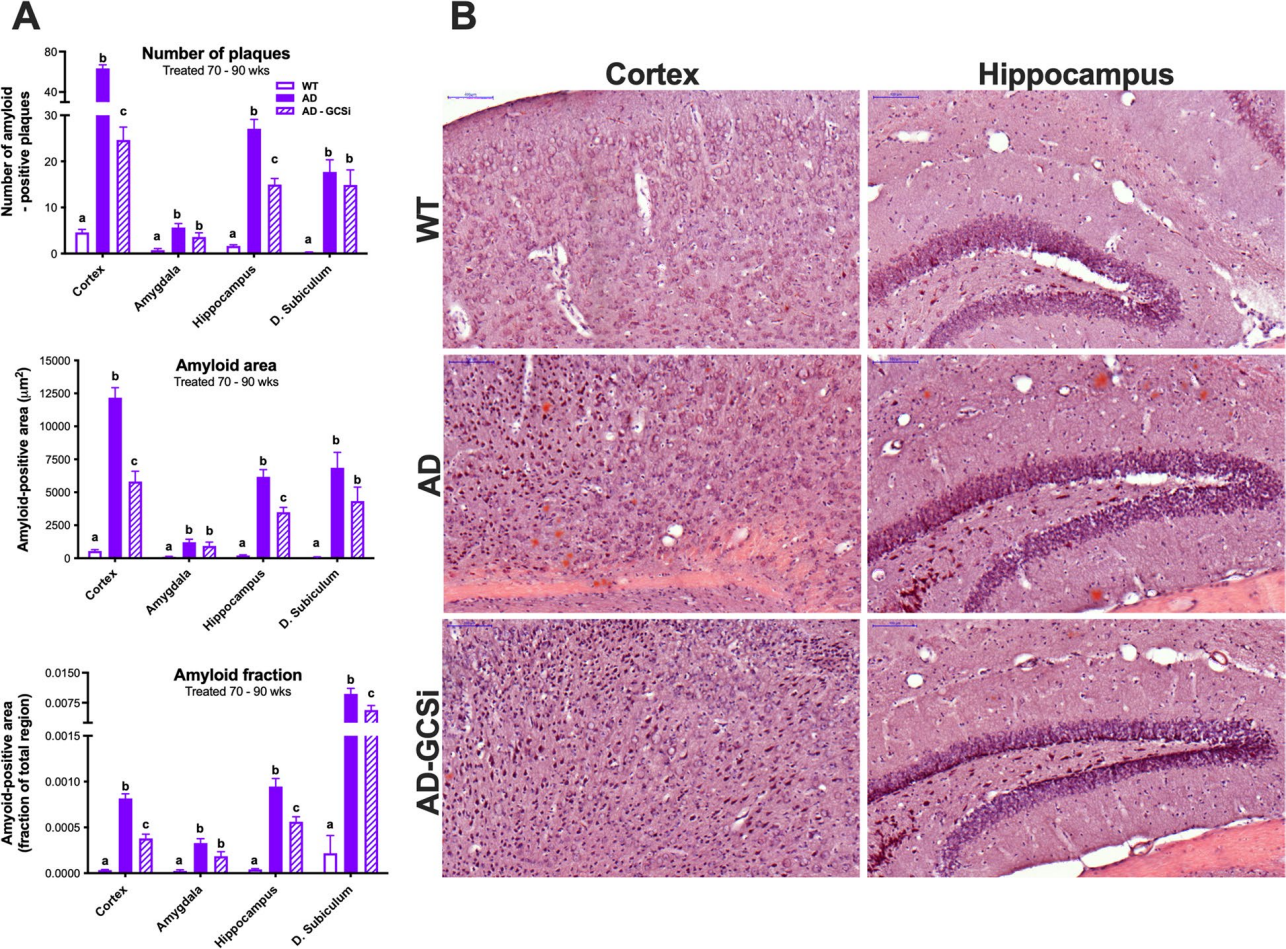
GCSi treatment reduces amyloid plaque burden in aged AD mice. **A** Amyloid plaque number, area, and fraction are significantly reduced in the cortex and hippocampus of AD (Tg2576) mice treated with a GCSi from 70 to 90 weeks of age. **B** 20X images of amyloid staining in the brains of WT-, AD-, and GCSi-treated AD mice (scale bar = 100 microns). See Figure S4 for 4X images (scale bar = 500 microns). Columns not connected by the same letter are significantly (*p* = 0.01) different from each other. Error bars represent ± SEM

Tg2576 mice feature age-related changes in amyloid beta (Aβ) and dense core plaque accumulation in the brain. Soluble Aβ is readily detected in young (i.e., 8 weeks old) Tg2576 mice; however, significant insoluble Aβ40 and Aβ42 accumulation does not manifest until 26–30 weeks of age. Notably, despite an exponential increase in Aβ accumulation after 34 weeks of age only a few dense core plaques are typically found in the brains of 44-week-old Tg2576 mice. However, between 52 and 66 weeks of age the Aβ plaque burden in Tg2576 mice approaches what is typically observed in the brains of AD patients [26]. In our first experiment, we determined whether GCSi treatment reduced Aβ accumulation in Tg2576 mice that were treated from 12 to 68 weeks of age. Data shown are collapsed across sexes because in our initial characterization studies we found that the rate of Aβ accumulation was equivalent between male and female Tg2576 mice. (Fig. S1). As expected, Aβ40/42 levels were significantly increased in the cortex, hippocampus, and amygdala of untreated Tg2576 mice compared to WT controls (Fig. 3, S3). In GCSi-treated Tg2576 mice, soluble Aβ42 was significantly reduced to WT levels in all three brain regions analyzed (Fig. 3A). GCSi treatment did not affect the levels of insoluble Aβ 42 or any Aβ 40 species (Fig. S3). Next, we evaluated Aβ neuropathology in Tg2576 mice that were treated with a GCSi from 70 to 90 weeks of age. Like our initial results, GCSi treatment lowered soluble Aβ42 levels in the brains of Tg2576 mice; however, the reduction was only significant for the hippocampus (Fig. 3B). Interestingly, amyloid burden (i.e., as assessed by the number of plaques, plaque area, and amyloid fraction) was significantly reduced both in the cortex and hippocampus, but not the amygdala of aged Tg2576 mice (Fig. 4, S4). Collectively, our results show that GCSi treatment significantly lowers the amyloid burden in the brain of AD model mice by slowing the accumulation rate of soluble Aβ42, an Aβ species that is particularly toxic to neurons [13] and a determinant of disease severity in AD patients [33].

### GCSi treatment affects remote memory consolidation in Tg2576 mice

**Fig. 5:**
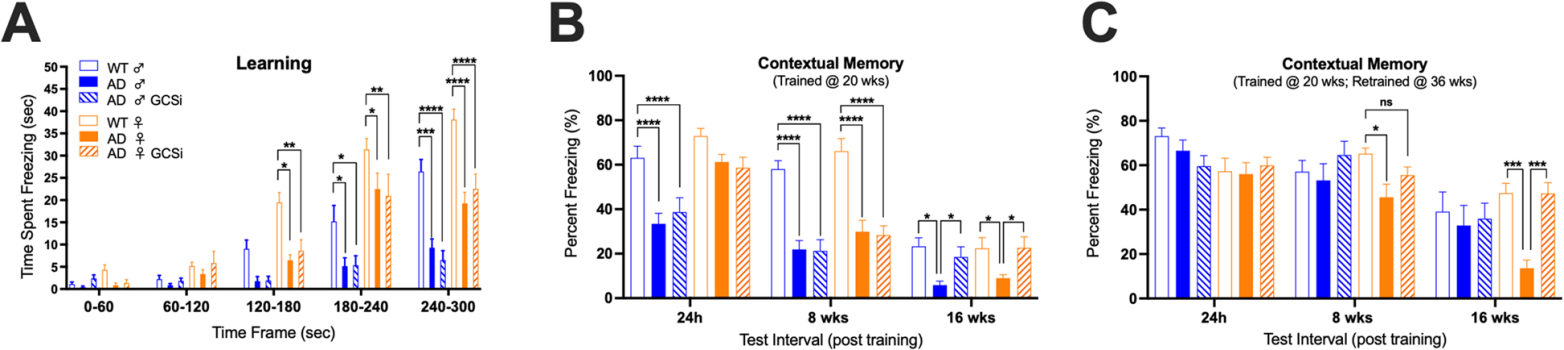
Remote memory consolidation in AD mice is significantly affected by GCSi treatment. **A** Learning and **B** contextual memory in AD (Tg2576) mice treated with a GCSi from 12 to 36 weeks of age. Mice were trained at 20 weeks of age. **C** Contextual memory in AD mice treated with a GCSi from 12 to 52 weeks of age that were re-trained at 36 weeks of age. Statistical comparisons for GCSi-treated AD mice are to sex matched wild type (WT) and to AD controls (****p = 0.0001, ***p = 0.001, **p = 0.01, and *p = 0.05). Error bars represent ± SEM

Next, we determined if GCSi treatment affected the manifestation of cognitive disease phenotypes in Tg2576 mice. Tg2576 mice fed either a GCSi formulated or a control base diet from 12 to 52 weeks of age were tested for deficits in learning, short-term memory, and remote memory consolidation using the contextual fear chamber conditioning test. Mice were initially trained and tested for short-term memory at 20 weeks of age. Remote memory consolidation was subsequently tested 8 (i.e., 28 weeks of age) and 16 (36 weeks of age) weeks later. Mice then underwent retraining (RT) and short-term memory re-testing at 36 weeks of age, and remote memory consolidation retesting 8 and 16 weeks later (i.e., at 44 and 52 weeks of age). GCSi treatment did not significantly improve learning or short-term memory performance in Tg2576 mice (Fig. 5A). In contrast, remote memory consolidation performance was similar between GCSi-treated mice and sex-matched WT controls 16 weeks post training (Fig. 5A). RT led to WT like short-term memory in both male and female Tg2576 mice regardless of treatment (Fig. 5B). Similarly, RT also led to WT like remote memory consolidation in male Tg2576 mice regardless of treatment (Fig. 5B). Notably, however, remote memory consolidation, which was still adversely affected in female Tg2576 mice, was now partially corrected in female GCSi treated mice 8 weeks post-RT (i.e., 44 weeks of age) and equivalent to similarly trained WT female mice 16-weeks post-RT (i.e., 52 weeks of age) (Fig. 5B). Collectively, our results suggest that ganglioside homeostasis is important for the establishment and maintenance of long-term memories.

## Discussion

Ganglioside homeostasis is essential for the normal development, maturation, health, and function of the CNS. For example, severe neurological disease manifests in individuals with extreme changes in ganglioside levels that are either due to a deficiency in ganglioside synthesis (e.g., epilepsy and hereditary spastic paraplegia) or hydrolysis (e.g., GM1 gangliosidosis, Tay-Sachs, and Sandhoff disease) [3, 20, 41, 43, 46]. Increasing evidence also suggests, however, that more subtle changes in ganglioside levels may contribute to the pathogenesis of more common neurological disorders including Parkinson disease, Huntington disease, amyotrophic lateral sclerosis, stroke, multiple sclerosis, and Alzheimer disease (AD) [15, 16, 47, 50]. Here, we show that pharmacological inhibition of glucosylceramide synthase (GCSi) in AD Tg2576 mice reduced aberrant GM3 ganglioside accumulation in the CNS, alleviated Aβ neuropathology, and improved some aspects of cognition.

Several studies indicate that ganglioside homeostasis is altered in post-mortem brain samples of AD patients [12, 36, 42], including a significant increase in GM3 levels in the cortex [8, 24, 28]. Elevated GM3 levels are particularly intriguing given that two large clinical studies (800+ patients/study) examining the peripheral lipidome of AD patients showed that increased GM3 levels were strongly associated with disease severity [22]. Like AD patients GM3 accumulation is also found in the brains of several AD rodent models including PS1, APP-PS1, 5xFAD, APP21, and TgCRND8 [7, 8, 27, 54]. Consistent with these results, in our current study, we found that GM3 levels were also significantly elevated both in the cortex and the amygdala of Tg2576 mice.

Genetic manipulation of ganglioside synthesis significantly affects Aβ neuropathology and cognitive phenotypes in multiple AD mouse models of disease [4, 17, 40]. For example, knockout of GD3 synthase reduced amyloid plaque levels and partially improved spatial learning in AD APP/PSEN1 mice [4]. As expected, removal of GD3 synthase in APP/PSEN1 mice eliminated b-series gangliosides (i.e., GD3 and its derivatives); however, it also led to an increase in the a-series gangliosides GM1 and GD1a. Interestingly, GM3, also an a-series ganglioside, was not affected in these mice, which suggests that it may also be regulated by an alternative pathway. Presumably, the reduced amyloid burden in GD3 deficient APP/PSEN1 mice was due to the lowering of b-series gangliosides, which are relatively enriched in sialic acid residues and fatty acids that favor the binding of Aβ [32, 39]. Reducing GD3 levels may have also slowed disease progression by promoting neuronal survival and reducing glial activation. GD3 is increased in many neurodegenerative conditions [47], accumulates in reactive astrocytes [25] and microglia [1], and induces apoptosis. Alternatively, the increase in GM1 and GD1a may have provided some neuroprotection [30, 35, 47] to improve cognition; however, this seems unlikely given that knockout of GM3 synthase in 5xFAD mice, which reduced both a and b-series gangliosides, also mitigated disease [17]. Interestingly, elimination of GM2 synthase, which promoted GM3 accumulation by blocking its metabolism to GM2, had the opposite effect on disease pathogenesis, and increased amyloid plaque burden in 1xFAD mice [40]. Although collectively these findings implicate ganglioside homeostasis as an important modulator of Aβ accumulation in vivo, they should be interpreted with caution; however, because ganglioside lowering in AD model mice was initiated during the embryonic development of the CNS. Here, in our current studies, we confirmed that glycosphingolipid homeostasis plays a central role in modulating Aβ neuropathology and cognitive function in AD mouse models. Importantly, we showed that GCSi lowered aberrant GM3 accumulation and mitigated Aβ neuropathology—even when treatment was initiated in aged Tg2576 mice. Notably, GCSi treatment reduced soluble Aβ42 levels, an Aβ species that is particularly toxic to neurons [13] and a determinant of disease severity in AD patients [33]. Furthermore, we also showed that GCSi treatment in aged Tg2576 mice led to a significant reduction in amyloid plaque burden in the hippocampus and cortex, but not in the amygdala. We also determined if early intervention with GCSi treatment prevented cognitive impairment in Tg2576 mice using the contextual fear chamber test. Although learning, cued (amygdala dependent) and short-term (hippocampal dependent) contextual cognition were not improved in GCSi treated Tg2576 mice, we did find that remote (cortical dependent) memory consolidation was significantly stabilized by treatment. Collectively, these findings indicate that glycosphingolipids are significant modulators of Aβ neuropathology and highlight a novel role for glycosphingolipids in being important for the consolidation of long-term memories.

### Limitations

Although our study was well planned, its interpretation has some limitations. For instance, cognitive function was only assessed using the contextual fear chamber test. Confirming the beneficial effects (or lack thereof) of GCSi on memory function in Tg2576 mice using additional cognitive tests would strengthen our conclusions. We planned to do this, however, in our initial behavioral characterization studies, we failed to detect cognitive dysfunction in 20- or 40-week-old Tg2756 mice (regardless of sex) using either the Barnes maze or novel object recognition tests. In addition, we only showed that GCSi significantly stabilized remote memory consolidation in Tg2576 mice when treatment was initiated prior to the onset of cognitive dysfunction. Showing that the reduction in Aβ neuropathology in aged GCSi-treated AD mice also lead to improved cognition would increase the likelihood of GCSi treatment being clinically translatable to AD patients. Our results also provide some insight into why developing Aβ modulating treatments for AD may be challenging. Notably, in multiple experiments, we found that Aβ levels were not the sole determinant of cognitive dysfunction in Tg2576 mice. First, in our characterization studies, we found that sex differences in memory impairment were not associated with sex differences in the rate of Aβ accumulation in the hippocampus, amygdala, or cortex. Future studies examining subcellular variations in Aβ accumulation in various brain regions in different cell types in male and female AD mice may yield alternative results. Second, enhancing cognitive training alone (even in mice with robust Aβ42 accumulation) was sufficient to mitigate the manifestation of some cognitive phenotypes in male, but not in female AD mice. This result also suggests female AD mice are relatively more vulnerable to disease, which is intriguing because women are at a higher risk for developing AD [19]. And third, reducing aberrant GM3 levels in the amygdala led to a concomitant reduction in soluble Aβ42 levels in GCSi-treated AD mice, but this did not improve cued (i.e., amygdala dependent) memory. Moreover, unlike short- and long-term contextual memory, amygdala dependent memory was not improved in male AD mice exposed to enhanced cognitive training. Furthermore, in our experiment that tested the efficacy of GCSi in aged AD mice amyloid plaque burden was not lowered in the amygdala. Thus, the amygdala may be particularly sensitive to Aβ-mediated toxicity. And lastly, GCSi treatment had no effect on disease related changes in learning or hippocampal dependent short-term memory in Tg2576 mice. This result may suggest that multiple therapeutic targets need to be engaged in parallel to improve the various aspects of cognitive decline in AD.

## Conclusions

In summary, the results of our experiments corroborate that GM3 accumulation is a manifestation of AD, that glycosphingolipids are important modulators of amyloid burden in the CNS and indicate glycosphingolipid homeostasis plays an important role in stabilizing long-term memories. Our findings also suggest that GCSi treatment in combination with therapeutic approaches that alleviate hippocampal and amygdala-dependent cognitive dysfunction may lead to a clinically meaningful treatment for AD.

## Supplementary Information

**Additional file 1: Figure S1.** AD mice do not display sex differences in the rate of oligomeric Aβ accumulation in the brain. (A) Soluble Aβ40 and insoluble (B) Aβ42 oligomeric species were measured in the amygdala, hippocampus, and cortex of male and female AD (Tg2576) mice at 20 and 36 weeks of age. Insoluble Aβ40 and Aβ42 oligomeric species were not detected in the brains of AD mice regardless of age and sex. Columns not connected by the same letter are significantly (*p* = 0.01) different from each other. Error bars represent ± SEM.

**Additional file 2: Figure S2.** GlcCer and GM1 levels in AD mice after GCSi treatment. (A) Serum GlcCer levels in AD (Tg2576) GCSi treated mice 1-week post treatment confirms compound activity. (B) Total GlcCer levels in the cortex and the hippocampus are reduced in AD after GCSi treatment. (C) Total ganglioside GM1 levels are unaffected by disease in the cortex, hippocampus, and amygdala and are not reduced following GCSi treatment. Columns not connected by the same letter are significantly (*p* = 0.01) different from each other. Error bars represent ± SEM.

**Additional file 3: Figure S3.** GCSi treatment does not affect insoluble Aβ40 and Aβ42 accumulation in the brains of AD mice. Insoluble Aβ40 and Aβ42 species in the amygdala, hippocampus, and cortex of AD (Tg2576) mice after GCSi treatment from (A) 12 to 68 weeks of age and (B) 70 to 90 weeks of age (BLOD, below the limit of detection). Columns not connected by the same letter are significantly (*p* = 0.01) different from each other. Error bars represent ± SEM.

**Additional file 4: Figure S4.** GCSi treatment reduces amyloid staining in aged AD mice. (A) 4X images of amyloid staining in the brains of WT, AD (Tg2576) and GCSi treated AD mice (scale bars = 500 microns).

**Additional file 5.**

## Data Availability

The datasets used and/or analyzed during the current study available from the corresponding author on reasonable request.

## Abbreviations

Aβ: Amyloid beta
Aβ42: Aβ with 42 amino acid peptides
Aβ40: Aβ with 40 amino acid peptides
AD: Alzheimer’s disease
APP: Amyloid precursor protein
CS: Conditioned stimulus
GlcCer: Glucosylceramide
GCSi: Glucosylceramide synthase inhibitor
MIT: More intense training
RT: Retrained
US: Unconditioned stimulus
