# Supplementary figures and images for: Glucosylceramide synthase inhibition reduces ganglioside GM3 accumulation, alleviates amyloid neuropathology, and stabilizes remote contextual memory in a mouse model of Alzheimer’s disease
Source: Alzheimers Res Ther. 2022 Feb 1;14:19. doi: 10.1186/s13195-022-00966-0 (PMC8805417; doi:10.1186/s13195-022-00966-0)

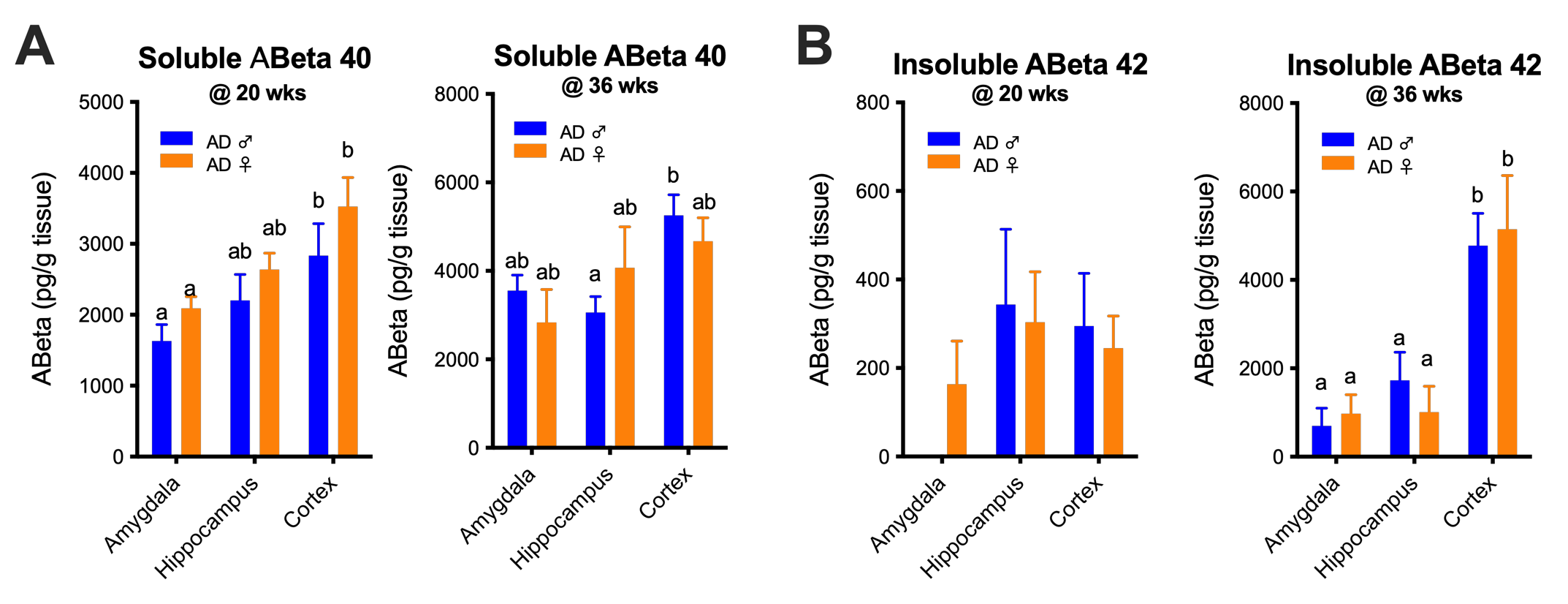

Supplement: Supplementary file 1 — Additional file 1: Figure S1. AD mice do not display sex differences in the rate of oligomeric Aβ accumulation in the brain. (A) Soluble Aβ40 and insoluble (B) Aβ42 oligomeric species were measured in the amygdala, hippocampus, and cortex of male and female AD (Tg2576) mice at 20 and 36 weeks of age. Insoluble Aβ40 and Aβ42 oligomeric species were not detected in the brains of AD mice regardless of age and sex. Columns not connected by the same letter are significantly (p = 0.01) different from each other. Error bars represent ± SEM. [file 13195_2022_966_MOESM1_ESM.tiff]

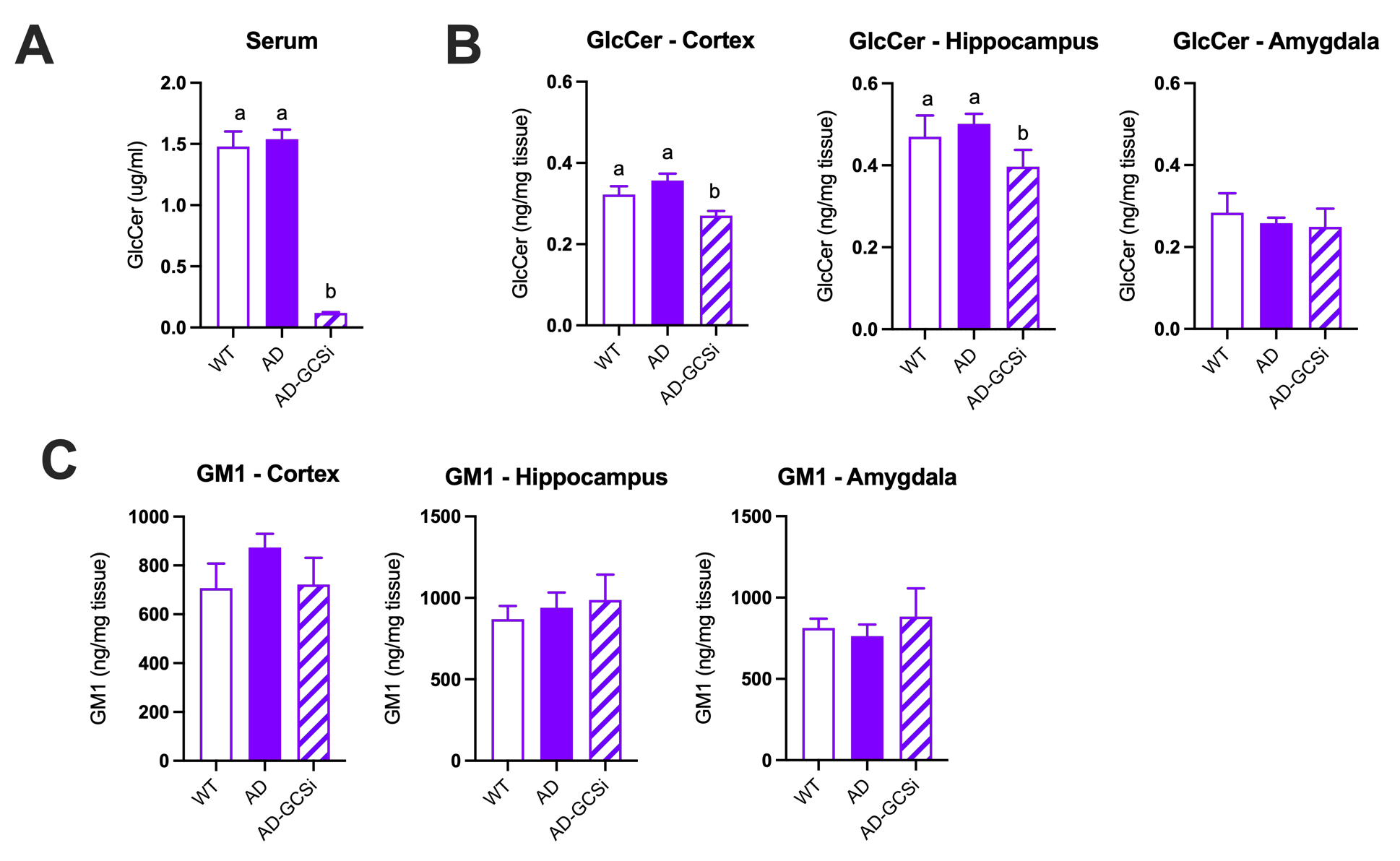

Supplement: Supplementary file 2 — Additional file 2: Figure S2. GlcCer and GM1 levels in AD mice after GCSi treatment. (A) Serum GlcCer levels in AD (Tg2576) GCSi treated mice 1-week post treatment confirms compound activity. (B) Total GlcCer levels in the cortex and the hippocampus are reduced in AD after GCSi treatment. (C) Total ganglioside GM1 levels are unaffected by disease in the cortex, hippocampus, and amygdala and are not reduced following GCSi treatment. Columns not connected by the same letter are significantly (p = 0.01) different from each other. Error bars represent ± SEM. [file 13195_2022_966_MOESM2_ESM.tiff]

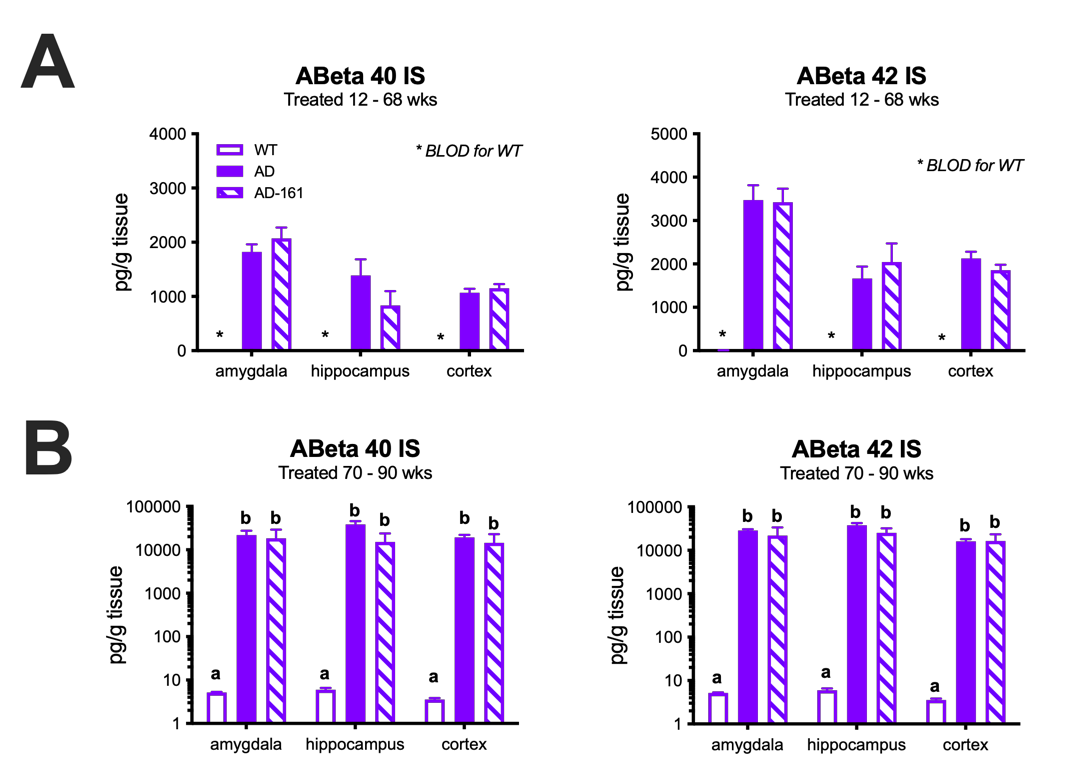

Supplement: Supplementary file 3 — Additional file 3: Figure S3. GCSi treatment does not affect insoluble Aβ40 and Aβ42 accumulation in the brains of AD mice. Insoluble Aβ40 and Aβ42 species in the amygdala, hippocampus, and cortex of AD (Tg2576) mice after GCSi treatment from (A) 12 to 68 weeks of age and (B) 70 to 90 weeks of age (BLOD, below the limit of detection). Columns not connected by the same letter are significantly (p = 0.01) different from each other. Error bars represent ± SEM. [file 13195_2022_966_MOESM3_ESM.tiff]

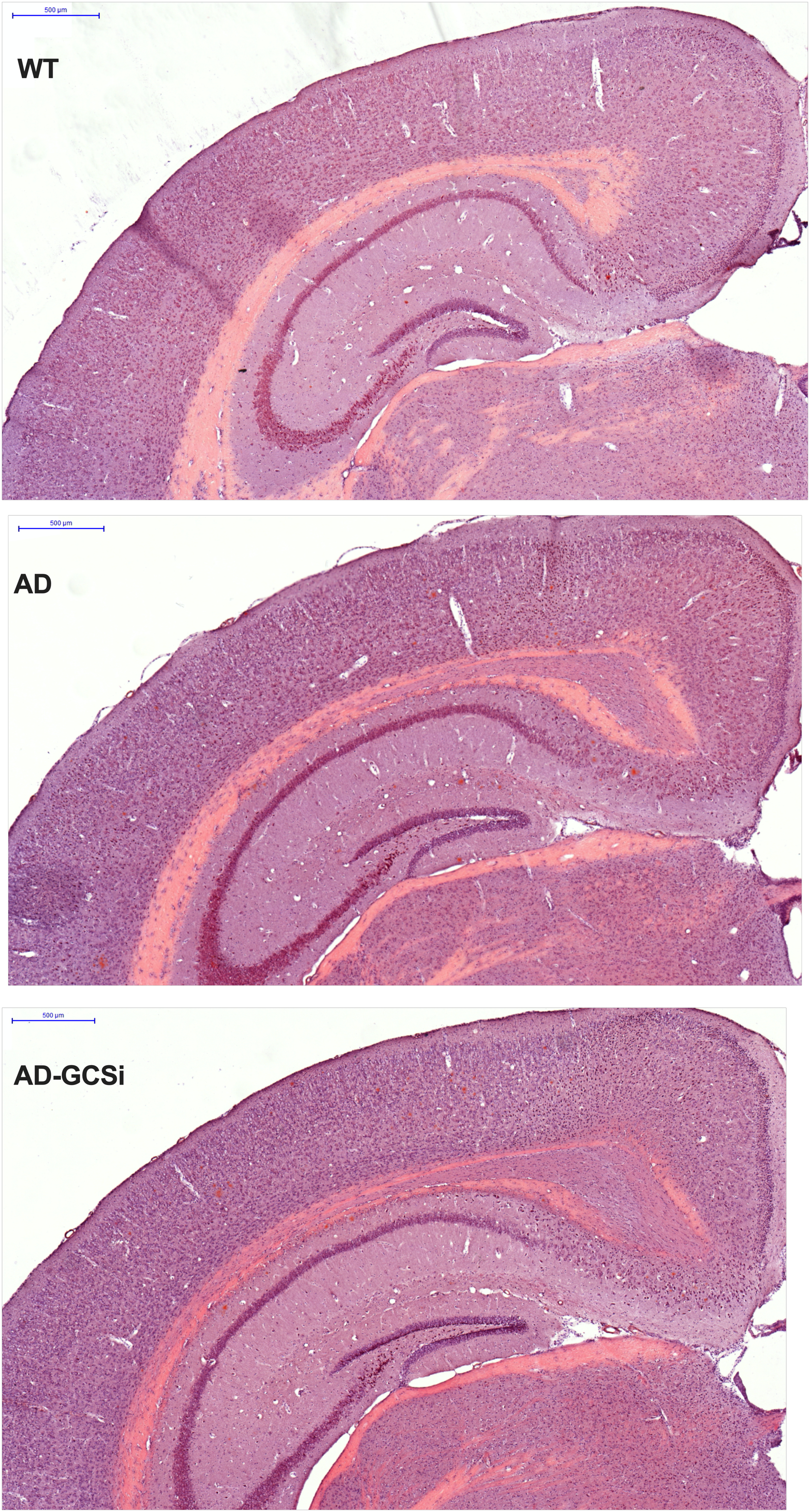

Supplement: Supplementary file 4 — Additional file 4: Figure S4. GCSi treatment reduces amyloid staining in aged AD mice. (A) 4X images of amyloid staining in the brains of WT, AD (Tg2576) and GCSi treated AD mice (scale bars = 500 microns). [file 13195_2022_966_MOESM4_ESM.tiff]
