# Supplementary material for: Glucosylceramide synthase inhibition reduces ganglioside GM3 accumulation, alleviates amyloid neuropathology, and stabilizes remote contextual memory in a mouse model of Alzheimer’s disease
Source: Alzheimers Res Ther. 2022 Feb 1;14:19. doi: 10.1186/s13195-022-00966-0 (PMC8805417; doi:10.1186/s13195-022-00966-0)
Supplement: Supplementary file 5 — Additional file 5. [file 13195_2022_966_MOESM5_ESM.docx]

| **Cohort** | **N =** | **Experiment** | **End Point** | **Study Parameters** | **Figure(s)** |
| --- | --- | --- | --- | --- | --- |
| 1 | 12/sex/genotype | Behavioral Characterization | Fear conditioning | - Trained (2x US-CS pairings) @ 12 wks.  - Tested @ 24h, 20 wks. & 28 wks. | 1 |
| 2 | 12/sex/genotype | Behavioral Characterization | Fear conditioning | - Trained (2x US-CS pairings) @ 20 wks.  - Tested @ 24h, 28 wks. & 36 wks. | 1 |
| 3 | 12/sex/genotype | Behavioral Characterization | Fear conditioning | - Trained (2x US-CS pairings) @ 36 wks.  - Tested @ 24h, 44 wks. & 36 wks. | 1 |
| 4 | 12/sex/genotype | Behavioral Characterization | Fear conditioning | - Trained (2x US-CS pairings) @ 12 wks.  - Tested @ 24h, 44 wks. & 36 wks.  - Retrained (2x US-CS pairings) @ 36 wks.  - Retested @ 24h, 44 wks. & 52 wks. | 1 |
| 5 | 12/sex/genotype | Behavioral Characterization | Fear conditioning | - Trained (4x US-CS pairings) @ 36 wks.  - Tested @ 24h, 44 wks. & 36 wks. | 1 |
| 6 | 6/sex/age | Aβ Characterization | **Aβ** ELISA | - Brain tissue collected @ 20 and 36 wks  - Analysis of amygdala, hippocampus & cortex | S1 |
| 7 | 4/sex/group | Glycosphingolipid levels after GCSi | LC/MS/MS | - Treated with GCSi from 12 - 68 weeks  - Analysis of amygdala, hippocampus & cortex | 2, S2 |
| 8 | 3-4/sex/group | Aβ levels after GCSi (early treatment) | **Aβ** ELISA | - Treated with GCSi from 12 - 68 weeks  - Analysis of amygdala, hippocampus & cortex | 3 |
| 10 | 3-4/sex/group | Aβ levels after GCSi (late treatment) | **Aβ** ELISA | - Treated with GCSi from 70 - 90 weeks  - Analysis of amygdala, hippocampus & cortex | 3, S3 |
| 11 | 2/sex/group | Amyloid burden after GCSi (late treatment) | Amyloid red stain | - Treated with GCSi from 70 - 90 weeks  - Analysis of amygdala, hippocampus & cortex | 4, S4 |
| 12 | 10/sex | Cognition after GCSi (early treatment) | Fear conditioning | - Treated with GCSi from 12 – 52 wks  - Trained (2x US-CS pairings) @ 20 wks.  - Tested @ 24h, 28 wks. & 36 wks.  - Retrained (2x US-CS pairings) @ 36 wks.  - Retested @ 24h, 44 wks. & 52 wks. | 5 |
